# Hormone Replacement Therapy and Cardiovascular Outcomes by Race and Ethnicity: MESA (Multi-Ethnic Study of Atherosclerosis)

**DOI:** 10.1016/j.jacadv.2025.102561

**Published:** 2026-01-23

**Authors:** Spencer Flynn, Amier Haidar, Icy Liang, Karol Watson, Tamara Horwich, Preethi Srikanthan

**Affiliations:** a Department of Medicine, Massachusetts General Hospital, Boston, Massachusetts, USA; b UCLA Department of Medicine, Los Angeles, California, USA; c Brown University, Providence, Rhode Island, USA; d The Warren Alpert Medical School of Brown University, UCLA Division of Cardiology, Los Angeles, California, USA; e UCLA Division of Cardiology, Los Angeles, California, USA; f UCLA Division of Endocrinology, Los Angeles, California, USA

**Keywords:** cardiovascular outcomes, ethnicity, hormone replacement therapy, race

## Abstract

**Background:**

There is mixed data regarding hormone replacement therapy (HRT) and cardiovascular disease (CVD), particularly on how timing of HRT initiation close to menopause may affect outcomes, and there is little data among different race/ethnicity groups.

**Objective:**

The purpose of this study was to how HRT use and cardiovascular outcomes differ by race/ethnicity.

**Methods:**

The Multi-Ethnic Study of Atherosclerosis is a prospective epidemiologic study of participants without CVD at enrollment. Outcomes were (1) all-cause mortality and (2) major adverse cardiovascular events (MACEs). Cox models were developed, focusing on the timing of HRT initiation and differences by race/ethnicity (White, Black, Hispanic, and Chinese).

**Results:**

There were 2,427 postmenopausal women with data on HRT and outcomes, followed up for a median of 14 years. HRT use within 5 years of menopause was associated with decreased MACE and all-cause mortality (HR: 0.72 [95% CI: 0.55-0.96] and HR 0.62 [95% CI: 0.48-0.80], respectively). These findings differed by racial/ethnic groups, with Chinese participants on HRT having increased MACE and a trend towards increased mortality (HR: 2.27 [95% CI: 1.06-4.87] and HR: 1.34 [95% CI: 0.73-2.47], respectively). These findings were only seen in Chinese participants who had the metabolic syndrome or elevated triglyceride levels.

**Conclusions:**

We found relative benefit with early initiation of HRT in all race/ethnic groups except Chinese, adding to the complex literature on HRT use in CVD primary prevention. However, Chinese women with the metabolic syndrome or elevated triglycerides may have increased risk of adverse cardiovascular outcomes with HRT, suggesting further research is needed on racial and metabolic differences in the cardiovascular impact of HRT use.

Given the cardiometabolic changes that occur during menopause, including increasing visceral fat mass, insulin resistance, and dyslipidemia, menopause is considered a female-specific risk factor for cardiovascular disease (CVD).^1^, ^2^, ^3^, ^4^, ^5^ Hormone replacement therapy (HRT) remains the most-effective treatment for the vasomotor symptoms that occur in over 75% of women during menopause.^6^ However, the relationship between menopause, HRT, and cardiovascular health is complex, and whether HRT may prevent vs accelerate CVD is debated.^7^

Early observational studies linked HRT with improved cardiovascular outcomes and mortality.^8^, ^9^, ^10^ However, the WHI (Women’s Health Initiative), a large randomized controlled trial, found an increased risk of coronary heart disease (CHD), stroke, venous thromboembolism, and breast cancer among a primary prevention cohort of women taking hormone replacement.^11^ Following these initial results from the WHI trial, clinical initiation of HRT declined to about a third of prior levels and has remained low.^7^^,^^12^^,^^13^ Subsequent studies have continued to find high breast cancer risks with HRT.^14^^,^^15^ However, re-evaluations of the WHI painted a more nuanced picture of CHD risk, with a finding that earlier initiation of HRT within 10 years of menopause was associated with better CHD outcomes and a favorable overall risk profile.^7^^,^^16^^,^^17^ Additional observational studies and randomized trial data have supported this “timing hypothesis” that early initiation of HRT closer to the onset of menopause, or before the age of 60, may confer cardiovascular benefits, whereas initiating HRT later may increase cardiovascular risk.^16^^,^^18^, ^19^, ^20^, ^21^, ^22^ The leading mechanistic theories for this timing hypothesis suggest that HRT’s actions may not be purely due to older age at HRT initiation, but rather the effect of chronologically cumulative atherosclerotic burden.^7^^,^^16^^,^^23^ Accordingly, it may be that risk factors for atherogenesis and inflammation determine who benefits from HRT. Notably, race and ethnicity are associated with many atherogenic risk factors, including obesity, waist circumference, lean mass, inflammatory biomarkers, and cholesterol burden, due to a mixture of genetic differences across populations as well as socioeconomic and cultural differences.^24^, ^25^, ^26^, ^27^

However, the role of race or ethnicity in HRT and cardiovascular outcomes is surprisingly poorly studied. This is in part because prior studies were predominantly conducted in the White population. For example, the WHI study population was 84% White, 6.8% Black, 5.3% Hispanic, and only 2.2% Asian. Another influential study on the effect of HRT on cardiovascular outcomes was conducted in Finland with a primarily White population.^28^ Furthermore, White women are more than twice as likely to receive HRT than Black women.^29^ The few studies that have investigated racial differences in HRT treatment and outcomes were either small and focused on only one racial group or focused primarily on oncologic outcomes rather than cardiovascular.^30^^,^^31^

The MESA (Multi-Ethnic Study of Atherosclerosis) is composed of a well-characterized, ethnically diverse population that is 38% White, 28% Black, 22% Hispanic, and 12% Chinese and has been rigorously followed in 7 examinations with yearly follow-up since July 2000. Hence, it is an ideal population to study the cardiovascular and mortality implications of HRT and the role of race/ethnicity. To our knowledge, the role of race and ethnicity on the association between HRT and cardiovascular outcomes and mortality has not been studied in a large, diverse sample such as MESA, and thus, this will be the objective of the present study.

## Methods

### Study design and population

MESA is a prospective, ongoing cohort study of 6,814 men and women, aged between 45 and 84 years at study baseline and without clinically apparent CVD. Participants were recruited via mailing lists, phone directories, and community events. Baseline data were collected from July 2000 and August 2002 (Examination 1) from the following 6 United States communities: Baltimore City and Baltimore County, Maryland; Chicago, Illinois; Forsyth County, North Carolina; Los Angeles County, California; Northern Manhattan and the Bronx, New York; and St. Paul, Minnesota. Participants were contacted every 9 to 12 months throughout the study to assess and adjudicate clinical morbidity and mortality. The study protocol was approved by the institutional review boards at all field centers. During the baseline examination, all participants provided written informed consent, and the participants were invited to complete subsequent exams and questionnaires. Further details of the study design have previously been described.^32^ Participants were asked about the use of HRT. In the present study, participants were required to be female, to have responded to the question about HRT use, and to have follow-up outcome data for at least 1 additional MESA examination. Premenopausal participants were excluded. Surgical menopause participants were also excluded as they were outliers at the time of initiating HRT (mean age of menopause: 43.9 years), and current guidelines recommend HRT use in individuals with surgical menopause before the age of 45 years.^33^ The study was approved by the institutional review boards of the participating institutions, and all participants provided informed consent.

### Covariates

Height was measured with a stadiometer and body weight with a balance scale to the nearest 0.1 cm and 0.5 kg, respectively. Waist circumference was measured using a steel measuring tape (standard 4-oz tension) from midway between the last rib and the superior border of the iliac crest during normal breathing and measured to the nearest 0.1 cm. Hip circumference was measured from the largest diameter of the hip and measured to the nearest 0.1 cm. Body mass index (BMI) was calculated by dividing weight in kg by height in meters squared. Seated blood pressure was measured using an automated cuff 3 times at 1-minute intervals following a standardized protocol. The average of the last 2 measurements was used for analysis. Standardized questionnaires were used to ascertain age, sex, race and ethnicity, education, income levels on a 1 to 13 scale as previously described,^32^ occupational information, smoking status, medical history, physical activity, and medication use for diabetes, lipid lowering, and hypertension. Total and high-density lipoprotein cholesterol and triglycerides were measured at a central laboratory (University of Vermont, Burlington, Vermont). Presence of the metabolic syndrome was ascertained according to the National Cholesterol Education Program 2004 guidelines and was applied equivalently across racial groups.^34^

### Menopause and HRT

MESA participants who both reported having undergone menopause and no menstrual periods for the last 12 months were considered to have undergone menopause. To ascertain HRT use, all MESA participants were asked if they were currently receiving HRT or had previously received HRT, the age they started on HRT, and for former users, the age at which they discontinued HRT. Age of menopause was ascertained by asking participants, “At what age did you go through menopause?” All data on menopause and HRT use was taken from MESA Exam 1. HRT use was not tracked consistently beyond Exam 4, and so continued use throughout the study period cannot be accurately reported. There were 1,159 women who had used HRT overall, with only 169 participants on HRT by Exam 4, a mean of 4.8 years after Exam 1, and only 43 were new users of HRT between Exam 1 and Exam 4. Accordingly, the majority on HRT had discontinued use within a few years of Exam 1, and there were few new starters.

### CVD events

The cohort was followed for incident CVD events and all-cause mortality for a median of 13.9 years (interquartile range [IQR] 11.3-16.5). At intervals of 9 to 12 months, a telephone interviewer contacted each participant to inquire about interim hospital admissions, cardiovascular outpatient diagnoses, and deaths. In order to verify self-reported diagnoses, copies of all death certificates and medical records for hospitalizations and outpatient cardiovascular diagnoses were obtained and reviewed. Two physicians, blinded to information from MESA examinations, independently reviewed and classified CVD events and assigned incidence dates. Reviewers classified myocardial infarction (MI) as definite, probable, or absent, based primarily on combinations of symptoms, electrocardiography, and cardiac biomarker levels. The reviewers classified deaths from CHD or CVD as present or absent based on hospital records and interviews with families. Definite fatal CHD required a MI within 28 days of death, chest pain within the 72 hours before death, or a history of CHD and the absence of a known nonatherosclerotic or noncardiac cause of death. Neurologists reviewed and classified stroke as present if there was a focal neurologic deficit lasting 24 hours or until death, with a clinically relevant lesion on brain imaging, and no nonvascular cause. Hard CHD events included nonfatal MI, resuscitated cardiac arrest, and death resulting from CHD. We defined major adverse cardiovascular events (MACE) as nonfatal MI, resuscitated cardiac arrest, coronary revascularization, definite or probable heart failure, stroke, or death resulting from CHD. Follow-up was from the baseline examination until the first CVD event, loss to follow-up, or death.

### Statistical analysis

All normally distributed continuous variables were expressed as means and standard deviations, with Student’s *t*-test used for comparisons of 2 groups or one-way analysis of variance for comparisons of multiple groups. Nonnormally distributed continuous variables were expressed as median (IQR) and used Kruskal-Wallis test for comparisons. All categorical variables were expressed as frequencies and percentages, with chi-square tests used for comparisons.

Multivariate Cox regression analyses were used to determine the associations between HRT use and CV outcomes and mortality, including among participants who started HRT within 5 years of menopause or more than 5 years after menopause. Cox models included the following covariates that were selected a priori: age, income level, smoking status, diabetes, hypertension, physical activity, BMI, and low-density lipoprotein. In addition, Cox models with race and ethnicity interaction terms and with stratification by race and ethnicity were generated. Exploratory Cox models also were generated using each of a marker of inflammation (C-reactive protein [CRP]), a marker of atherosclerosis (coronary artery calcium score [CAC]), and the metabolic syndrome and included interaction terms between these variables and HRT. For all Cox models, proportional hazards assumption was verified through inspection of graphs of the Schoenfeld residuals. Cox models in the general cohort always used the never HRT group as the comparator. Cox models stratified by race/ethnicity always used the never HRT users within that race/ethnicity as the comparator. As a sensitivity analysis, multiple imputation by chained equations was performed to account for missing covariate data using twenty imputed datasets generated with predictive mean matching for continuous variables and appropriate logistic or multinomial regression models for categorical variables. Cox proportional hazards models were fit separately within each imputed dataset, and results were combined using Rubin’s rules. R (v. 4.3.2) was used to perform the statistical analysis, with the *survival* package (v 3.8-3) used for Cox regressions, and *mice* (v 3.16.0) used for imputations.

## Results

Descriptive characteristics of MESA exam 1 participants are displayed in Table 1. There were 1,159 women who had used HRT, while 1,268 women had not used HRT (see study flowchart, Supplemental Figure 1). The types of HRT included estrogen only (34.7%), estrogen and progesterone (29.0%), and not reported (36.3%). Formulation of hormone replacement (oral vs patch) was not specified. The majority of women on HRT (64.8%) started within 5 years of menopause, with median years of HRT use of 5 (IQR 11), and 60.1% of participants who had used HRT were still using HRT at the time of Exam 1. White women made up the majority (51.4%) of women who received HRT, while only accounting for 26.7% of women not on HRT (*P* for the difference among racial groups <0.001). Those on HRT differed significantly from those not on HRT across many variables, including age, BMI, waist circumference, waist hip ratio, systolic blood pressure, low-density lipoprotein, high-density lipoprotein, CRP, and income (see Table 1).

**Table 1:** Demographics and Clinical Characteristics of Participants Stratified by Use of Hormone Replacement Therapy

	No Hormone Replacement (n = 1,268)	Received Hormone Replacement (n = 1,159)	*P* Value
Age, y	65.5 (9.4)	63.3 (8.7)	<0.001
Age started hormone replacement	NA	52.2 (8.6)	NA
Using HRT at Exam 1	NA	697 (60.1%)	NA
Using HRT at Exam 4	NA	169 (14.6%)	NA
Years of HRT by Exam 1, median (IQR)	NA	5.0 (1-12.0)	<0.001
Race or ethnicity			<0.001
White	338 (26.7%)	596 (51.4%)	
Chinese	202 (15.9%)	102 (8.8%)	
Black	389 (30.7%)	259 (22.3%)	
Hispanic	339 (26.7%)	202 (17.4%)	
Weight, kg	73.0 (17.1)	72.3 (15.6)	0.304
Body mass index	29.1 (6.3)	28.0 (5.6)	<0.001
Waist circumference, cm	98.7 (15.8)	95.5 (15.1)	<0.001
Waist hip ratio	0.92 (0.08)	0.89 (0.08)	<0.001
Systolic blood pressure, mm Hg	131.3 (23.9)	126.4 (22.4)	<0.001
Low-density lipoprotein, mg/dL, median (IQR)	120.0 (99.0-142.0)	113.0 (94.0-134.0)	<0.001
High-density lipoprotein, mg/dL, median (IQR)	51.5 (44.0-62.0)	57.0 (47.0-68.0)	<0.001
Triglycerides, mg/dL, median (IQR)	111.0 (80.5-157.0)	115.0 (79.0-163.0)	0.213
C-reactive protein, mg/dL, median (IQR)	2.3 (1.0, 4.9)	2.9 (1.1, 5.9)	0.002
Creatinine, mg/dL, median (IQR)	0.8 (0.7-0.9)	0.8 (0.7-0.9)	0.90
Coronary artery calcium, Agatston, median (IQR)	0.0 (0.0-62.9)	0.0 (0.0-32.7)	<0.001
Hypertension	662 (52%)	550 (47%)	0.022
Diabetes, yes	152 (12%)	104 (9.0%)	0.019
Metabolic syndrome, yes	569 (45%)	415 (36%)	<0.001
Prior pregnancy, yes	1,125 (88.7%)	1,016 (87.7%)	0.455
Cancer, yes	112 (8.8%)	107 (9.2%)	0.80
Smoking			<0.001
Previous	317 (25%)	406 (35%)	
Current	132 (10%)	127 (11%)	
Exercise, MET minutes per week, median (IQR)	347 (1,53-660)	368 (1,98-645)	0.013
Prior oophorectomy	132 (11%)	228 (20%)	<0.001
Prior hysterectomy	272 (22%)	427 (37%)	<0.001
Income scale	6.8 (3.5)	8.6 (3.5)	<0.001
Hormone replacement type	NA		NA
Estrogen only		402 (34.7%)	
Estrogen and progesterone		336 (29.0%)	
Not recorded		421 (36.3%)	
MACE or cardiac death	195 (15%)	127 (11%)	0.002
All-cause mortality,	271 (21%)	166 (14%)	<0.001
Cardiac death	63 (5.0%)	32 (2.8%)	0.007
MACE, excluding cardiac death	170 (13%)	111 (9.6%)	0.004
Myocardial infarction	54 (4.3%)	30 (2.6%)	0.033
Stroke	69 (5.4%)	39 (3.4%)	0.017

All numeric variables are mean (SD) unless otherwise specified. Selected biomarkers are presented as median (IQR). All categorical variables are presented as count (%).

HRT = hormone replacement therapy; IQR = interquartile range; MACE = major adverse cardiovascular events; MET = metabolic equivalent of task.

### HRT use, timing, and outcomes

**Figure 1:**
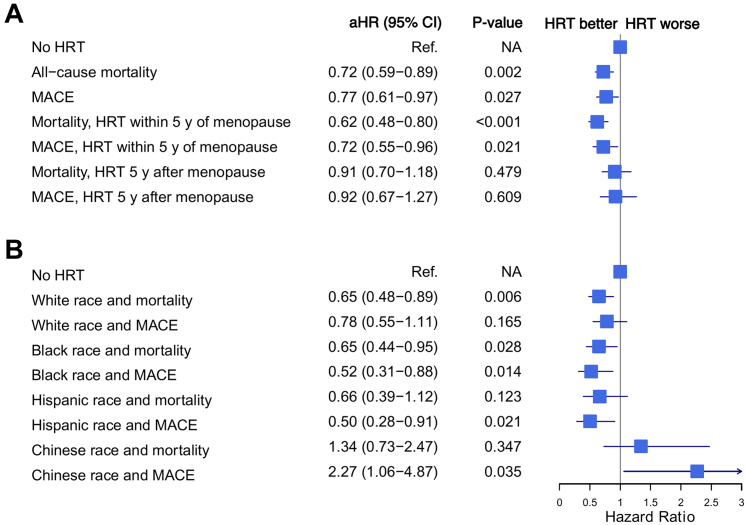
**Forest Plot of Outcomes Among Groups on Hormone Replacement Therapy** Each hazard ratio is from a separate Cox model. All models controlled for age, income level, BMI, smoking status, diabetes, hypertension, physical activity, and LDL levels and included HRT as the independent variable. MACE was defined as death from cardiac cause, resuscitated cardiac arrest, myocardial infarction, stroke, new heart failure diagnosis, or any coronary revascularization. Reference group for (A) was never HRT users in the full cohort, and that in (B) was never HRT users within the respective race/ethnicity groups. (A) Associations between HRT and outcomes within the full cohort, and by whether HRT was started within 5 years of menopause or more than 5 years after menopause. (B) Associations between HRT and outcomes by race and ethnicity. BMI = body mass index; HRT = hormone replacement therapy; LDL = low-density lipoprotein; MACE = major adverse cardiovascular events.

HRT use and its association with all-cause mortality or MACE are presented in Figure 1A. HRT use was significantly associated with a lower risk of both MACE (HR: 0.77, 95% CI: 0.61-0.97, *P* = 0.027) and all-cause mortality (HR: 0.72, 95% CI: 0.59-0.89, *P* = 0.002) in the fully adjusted Cox models. When MACE was added as a covariate to the mortality model, the significant association between HRT use and mortality risk persisted (HR: 0.75 [95% CI: 0.61-0.92], *P* = 0.006). Figure 1A) also presents the association between timing of HRT initiation and MACE/death. Earlier HRT initiation was associated with decreased risk of all-cause mortality (HR: 0.62, 95% CI: 0.48-0.80, *P* < 0.001), while women who had started HRT 5 years after menopause did not have decreased risk (HR: 0.91, 95% CI: 0.70-1.18), *P* = 0.479. Women who had started HRT within 5 years of menopause also had significantly lower risk of MACE (HR: 0.72, 95% CI: 0.55-0.96, *P* = 0.021) than those not on HRT, while women who started 5 years after menopause did not (HR: 0.92, 95% CI: 0.67-1.27, *P* = 0.610). A sensitivity analysis was performed excluding women who were within 5 years of menopause given that these women by definition could not be assigned to the late HRT initiation group, with similar findings (Supplemental Table 1). An additional sensitivity analysis by category of MACE (eg, MI, coronary revascularization, heart failure, stroke, and cardiovascular death) was also performed, with numeric associations favoring cardioprotection for HRT use (Supplemental Table 2). Early initiation of HRT was particularly associated with lower risk of stroke and cardiac-reason-related mortality in these subgroups. A sensitivity analysis stratified by median age of menopause onset did not find significant differences by age of onset with HRT use and outcomes. There was a numerical trend towards greater reduction in cardiac risk with early initiation of HRT within 5 years of menopause in the below-median age of menopause group compared to above-median age of menopause, but the interaction terms were nonsignificant (Supplemental Table 3). A final sensitivity analysis using the imputed dataset had similar findings to the primary analysis (Supplemental Tables 4 and 5).

### HRT, race and ethnicity, and outcomes

There were no significant interaction terms between race and ethnicity and HRT with MACE or all-cause mortality among White, Black, or Hispanic groups (*P*-values for interaction terms all >0.05), although there was a significant interaction term of Chinese ethnicity with both MACE and cardiac death (HR: 2.46, 95% CI: 1.11-5.44, *P* = 0.027, and HR: 2.18, 95% CI: 1.13-4.24, *P* = 0.021, respectively). Stratification by race and ethnicity was performed. Figure 1B presents the associations between HRT, MACE, and all-cause mortality stratified by race and ethnicity. No significant association existed between HRT and MACE among White women, while White women on HRT had a significantly lower risk of all-cause mortality (HR: 0.65, 95% CI: 0.48-0.89, *P* = 0.006). Black women on HRT had a significantly lower risk of both MACE (HR: 0.52, 95% CI: 0.31-0.88, *P* = 0.014) and all-cause mortality (HR: 0.65, 95% CI: 0.44-0.95, *P* = 0.028). Hispanic women on HRT had a significantly lower risk of MACE (HR: 0.50, 95% CI: 0.28-0.91, *P* = 0.021), but did not have a significantly lower risk of all-cause mortality.

The results for Chinese women diverged from those of the other racial groups. Chinese women who had used HRT had a significantly increased risk of MACE (HR: 2.27, 95% CI: 1.06-4.87, *P* = 0.035), compared to Chinese women who had not been on HRT. Chinese women on HRT also had an increased risk of all-cause mortality, although this was nonsignificant (HR: 1.34, 95% CI: 0.73-2.47, *P* = 0.347).

**Figure 2:**
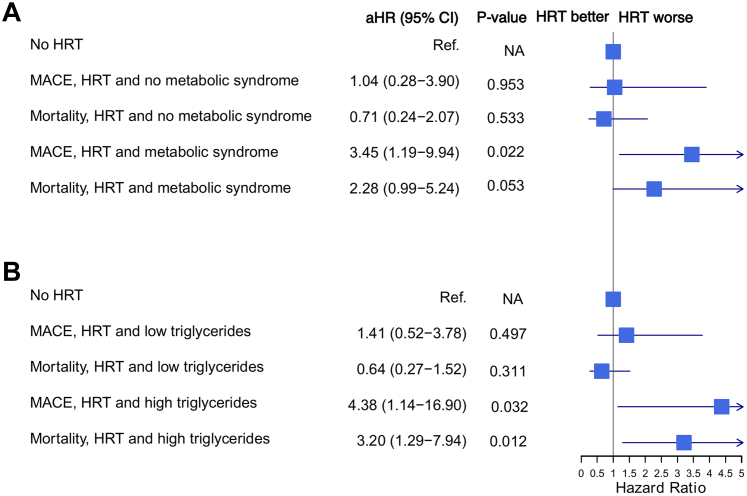
**Outcomes Among Chinese Participants on Hormone Replacement Therapy** Stratified by (A) the presence of the metabolic syndrome, or (B) triglycerides ≥150 mg/dL. Each hazard ratio depicted is from a separate Cox model. All models controlled for age, income level, BMI, smoking status, diabetes, hypertension, physical activity, and LDL levels and included HRT as the dependent variable. The reference group was never HRT users among the Chinese group. N = 193 for no metabolic syndrome, of whom 67 had used HRT, and N = 111 for the presence of the metabolic syndrome, of whom 37 had been on HRT. N = 108 with high triglyceride levels, of whom 31 had used HRT, and N = 196 for low triglycerides, of whom 71 had used HRT. The upper confidence intervals are clipped at HR 5.0 to fit within the plot.

Exploratory analyses were conducted to identify possible underlying mediators for these findings in the Chinese ethnic group. Notably, compared to other race and ethnicity groups, Chinese participants started HRT later after menopause (median 3 years after menopause compared to 1 year in the White group and 1-2 years in Black and Hispanic groups, respectively, see Table 2). While, in all racial groups besides Chinese, earlier initiation of HRT within 5 years of menopause led to lower hazard ratios for all-cause mortality or MACE, the association of Chinese ethnicity and HRT with higher MACE and death remained consistent in participants who received HRT within 5 years of menopause (Table 3). CRP levels, CAC, and body composition markers were also significantly different in Chinese ethnicity compared to those in other race and ethnic groups (Table 2), but these variables did not attenuate the association (see Supplemental Table 5). However, there was a significant interaction between Chinese ethnicity and HRT use with MACE by the presence of the metabolic syndrome (*P* interaction = 0.040). When stratified by the presence of the metabolic syndrome, Chinese participants with the metabolic syndrome had a significant association between HRT and increased MACE compared to Chinese women without the metabolic syndrome (see Figure 2A); HR: 3.45, 95% CI: 1.19-9.94, *P* = 0.022 vs HR: 1.04, 95% CI: 0.28-3.90, *P* = 0.953, respectively), with a borderline significant association with all-cause mortality (HR: 2.28, (n 0.99-5.24, *P* = 0.053 vs HR: 0.71, (n 0.24-2.07, *P* = 0.533), while those without the metabolic syndrome did not have increased risk of MACE or mortality. Similarly, when stratified by triglycerides ≥150 mg/dL, only individuals with high triglycerides had significant MACE (HR: 4.38, (n 1.14-16.9, *P* = 0.032 vs HR: 1.40, (n 0.52-3.78, *P* = 0.501, high vs low triglycerides, respectively) and all-cause mortality (HR: 3.20, (n 1.29-7.94, *P* = 0.012 vs HR: 0.64, (n 0.27-1.52, *P* = 0.31) (Figure 2B). There was a significant interaction term by triglycerides with all-cause mortality (*P* = 0.022). Other races/ethnic groups did not have significant interaction terms with presence of the metabolic syndrome, elevated triglycerides, or elevated BMI (Supplemental Table 6).

**Table 2:** Demographics and Clinical Characteristics of Participants Who Received Hormone Replacement Therapy by Race and Ethnicity

	White (n = 596)	Chinese (n = 102)	Black (n = 259)	Hispanic (n = 202)	*P* Value
Age, y	63.7 (8.8)	63.9 (8.9)	63.0 (8.1)	62.2 (8.9)	0.146
Weight, kg	71.5 (15.2)	57.3 (8.3)	80.5 (15.2)	71.8 (13.5)	<0.001
Body mass index	27.0 (5.5)	23.9 (3.1)	30.4 (5.6)	29.6 (5.1)	<0.001
Waist circumference, cm	93.9 (15.3)	85.9 (10.4)	99.7 (14.9)	99.5 (13.7)	<0.001
Waist hip ratio	0.88 (0.08)	0.90 (0.07)	0.89 (0.08)	0.94 (0.07)	<0.001
Systolic blood pressure, mm Hg	123.7 (21.4)	125.2 (21.1)	132.5 (22.9)	127.1 (23.8)	<0.001
Low-density lipoprotein, mg/dL, median (IQR)	111.0 (92.0-132.0)	110.5 (92.0-131.0)	119.0 (98.0-138.0)	117.0 (94.0-136.0)	0.056
High-density lipoprotein, mg/dL, median (IQR)	60.0 (49.0-71.0)	53.0 (46.0-62.0)	58.0 (48.0-67.0)	52.0 (44.0-63.0)	<0.001
Triglycerides, mg/dL, median (IQR)	117.5 (78.0-170.0)	125.0 (97.0-162.0)	91.0 (70.0-125.0)	135.0 (101.0-186.0)	<0.001
Coronary artery calcium, Agatston, median (IQR)	0.0 (0.0-47.6)	0.0 (0.0-55.1)	0.0 (0.0-13.8)	0.0 (0.0-14.7)	<0.001
Income scale	9.8 (3.0)	7.3 (3.8)	8.2 (3.3)	5.9 (3.2)	<0.001
C-reactive peptide, mg/dL, median (IQR)	2.9 (1.1-5.9)	0.9 (0.6-1.9)	3.4 (1.5-7.4)	3.5 (1.5-6.7)	<0.001
Creatinine, mg/dL, median (IQR)	0.8 (0.8-0.9)	0.7 (0.7-0.8)	0.9 (0.8-1.0)	0.8 (0.7-0.9)	<0.001
Diabetes present, yes	29 (4.9%)	11 (11%)	34 (13%)	30 (15%)	<0.001
Metabolic syndrome, yes	190 (32%)	35 (34%)	95 (37%)	95 (47%)	<0.001
Smoking					<0.001
Previous	268 (45%)	3 (2.9%)	89 (34%)	50 (25%)	
Current	64 (11%)	1 (1.0%)	39 (15%)	23 (11%)	
Exercise, MET minutes per week, median (IQR)	369 (218-593)	261 (105-435)	450 (216-820)	347 (170-688)	<0.001
Prior oophorectomy, yes	118 (20%)	10 (9.8%)	64 (26%)	36 (18%)	0.005
Prior hysterectomy, yes	196 (33%)	14 (14%)	141 (54%)	76 (38%)	<0.001
MACE or cardiac death	80 (13%)	13 (13%)	19 (7.3%)	15 (7.4%)	0.018
MACE	74 (12%)	11 (11%)	12 (4.6%)	14 (6.9%)	0.002
All-cause mortality	91 (15%)	17 (17%)	38 (15%)	20 (9.9%)	0.20
Cardiac death	16 (2.7%)	5 (4.9%)	7 (2.7%)	4 (2.0%)	0.50
Myocardial infarction	18 (3.0%)	6 (5.9%)	3 (1.2%)	3 (1.5%)	0.048
Stroke	25 (4.2%)	2 (2.0%)	5 (1.9%)	7 (3.5%)	0.30
Born in United States, yes	555 (93.1%)	4 (3.9%)	246 (95.0%)	66 (32.7%)	<0.001
Prior cancer, yes	83 (14%)	3 (2.9%)	9 (3.5%)	12 (5.9%)	<0.001
Years HRT use at Exam 1	9.1 (8.4)	4.3 (5.6)	7.4 (7.8)	5.3 (7.1)	<0.001
Age of menopause, median (IQR)	50 (45-52)	50 (48-52)	48 (41-52)	47 (42-50)	<0.001
Age started HRT, median (IQR)	50 (50-55)	53 (50-60)	49 (48-55)	49 (47-56)	<0.001
Started HRT within 5 years of menopause	418 (70.1)	57 (55.9%)	158 (61.0%)	118 (58.4%)	<0.001

All numeric variables are presented as mean (SD) unless otherwise specified. All categorical variables are presented as count (percentage).

HRT = hormone replacement therapy; IQR = interquartile range; MACE = major adverse cardiovascular events; MET = metabolic equivalent of task.

**Table 3:** Hormone Replacement Within 5 Years of Menopause and MACE/Death With Stratification by Race and Ethnicity

	White	Black	Hispanic	Chinese
MACE, within 5 y	0.74 (0.50-1.10) 0.136	0.55 (0.29-1.03) *P* = 0.062	0.37 (0.16-0.84) *P* = 0.018	2.17 (0.70-6.72) *P* = 0.179
MACE, after 5 y	0.93 (0.577-1.51) *P* = 0.779	0.61 (0.26-1.43) *P* = 0.258	0.75 (0.35-1.59) *P* = 0.456	2.04 (0.82-5.09) *P* = 0.126
All-cause mortality, within 5 y	0.64 (0.45-0.91) *P* = 0.013	0.61 (0.38-0.98) *P* = 0.041	0.28 (0.11-0.71) *P* = 0.008	1.33 (0.52-3.41) *P* = 0.549
All-cause mortality, after 5 y	0.70 (0.46-1.08) *P* = 0.106	0.68 (0.36-1.29) *P* = 0.240	1.26 (0.70-2.26) *P* = 0.444	1.39 (0.69-2.82) *P* = 0.355

Values are HR (95% CI). All models included age, smoking status, diabetes, BMI, hypertension, physical activity, income, and LDL levels as covariates. Reference group is no HRT within the specified race/ethnic group for each model.

BMI = body mass index; HRT = hormone replacement therapy; LDL = low-density lipoprotein; MACE = major adverse cardiovascular event.

## Discussion

The present study examined the associations between HRT use with CVD and mortality in a large, ethnically diverse population of the MESA study. We found HRT use initiated within 5 years of menopause was associated with lower MACE and all-cause mortality. This finding was noted in all races except among Chinese women who had a higher risk of MACE regardless of timing of HRT initiation (Central Illustration). This increased risk of MACE with HRT use among Chinese women appeared to be only among those with the metabolic syndrome or with elevated triglycerides.

**Central Illustration:**
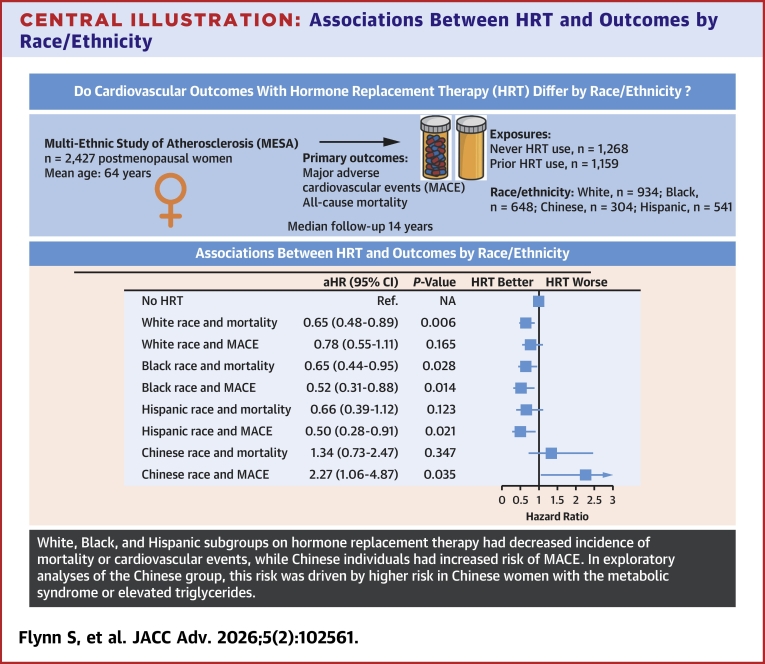
**Associations Between HRT and Outcomes by Race/Ethnicity** Reference group is never HRT users within the same race or ethnic group. Models adjusted for age, income level, BMI, smoking status, diabetes, hypertension, physical activity, and LDL levels. Pinteraction for Chinese race <0.05 for both MACE and mortality.

We found that in the full cohort, HRT use within 5 years of menopause was associated with lower MACE and all-cause mortality. The relationship between earlier timing of HRT use and better cardiovascular outcomes was initially observed in secondary analyses of the WHI randomized controlled trial, which showed that women who initiated hormone therapy closer to menopause tended to have reduced CHD risk compared with an increase in CHD risk among women more distant from menopause.^35^ Since then, several studies have supported the timing hypothesis, showing that earlier HRT use may reduce mortality and cardiovascular events.^7^^,^^19^^,^^36^^,^^37^ The exact mechanism for the timing hypothesis has not been elucidated although pathophysiologic pathways have been investigated. HRT may prevent the development of atherosclerosis in those with healthy blood vessels, while promoting plaque rupture, thrombosis, and inflammation in those with significant atherosclerosis. The ELITE (Early versus Late Intervention Trial with Estradiol) randomized controlled trial provides evidence for this theory.^22^ It found that women who were randomized to HRT within 5 years of menopause had lower rates of carotid plaque atherosclerosis than placebo, while women who initiated HRT 10 years after menopause had similar rates of atherosclerosis progression compared to placebo. Mechanistic studies also suggest that in young women without significant atherosclerosis, estrogen increases nitric oxide synthesis and vasodilation and decreases inflammatory cell adhesion, which overall slows the generation of atherosclerosis. However, in older women with significant plaques, it is believed that estrogen receptors are less functional, and so these benefits do not occur, and instead estrogen predisposes existing plaque to rupture and thrombosis.^7^^,^^16^^,^^23^

Intriguingly, the association with decreased all-cause mortality in those who initiated HRT within 5 years of menopause was stronger than with MACE. In addition, when MACE was added as a covariate in Cox models of all-cause mortality, the use of HRT remained as a significant predictor, indicating that some of the prevention measures of all-cause mortality may have been independent of cardiovascular benefit. We do not have data to better elucidate what the other causes of death beyond cardiovascular death may have been, but it is notable that in the WHI, the use of HRT was associated with lower fracture risk and colorectal cancer, both of which are common causes of death in older women.^11^^,^^38^^,^^39^ HRT also may have beneficial effects on body composition, through altered visceral fat mass and muscle mass accrual and insulin sensitivity.^40^^,^^41^

We found that HRT use was associated with increased adverse cardiovascular outcomes in Chinese women. To our knowledge, this finding has not been shown before. Interestingly, two recent population-based cohort studies using insurance data in Korea and Taiwan both found an increased risk of CVD among women using HRT, including increased risks of both stroke and coronary disease.^42^^,^^43^ This is notably different from similar studies in predominately White populations in the United States and Europe, which have generally found reduced CVD in retrospective studies of HRT use.^8^^,^^44^ Notably, our Chinese participants were almost entirely immigrants to the United States (11 out of 304 were born in the United States), which may lead to unmeasured confounding or indicate unique behaviors within that subpopulation. Given our small sample size of Chinese women, our findings should be interpreted as hypothesis-generating and spur further investigation.

We conducted exploratory analyses to identify whether known or hypothesized mediators of the effects of HRT on cardiovascular mortality might underly the finding of increased CVD risk in Chinese women on HRT. Most intriguingly, timing of initiation did not mediate the finding. Additional analyses also did not find significant interactions with a measure of inflammation (CRP), or a proxy measure of coronary atherosclerosis (CAC). However, there was a positive interaction with metabolic syndrome or high triglycerides and Chinese ethnicity with HRT use. Thus, Chinese women with the metabolic syndrome who were taking HRT had a significantly increased risk of MACE, while Chinese women without the metabolic syndrome did not have an increased risk of MACE, with borderline significant results with total mortality as well. Similarly, Chinese women on HRT with triglyceride levels ≥150 had significantly higher rates of MACE and all-cause mortality. Interestingly, the WHI also found that participants with metabolic syndrome on HRT had over double the risk of cardiovascular events compared to those without metabolic syndrome on HRT.^45^ In our study, we only find this association with Chinese participants and not in other racial and ethnic groups, and we additionally found that of all components of the metabolic syndrome, it appears that triglyceride levels drove the increased risk.

This unique finding among Chinese participants should be interpreted as exploratory and hypothesis generating. It supports the theory that risk factors for atherogenesis and inflammation may be important in determining benefit from HRT, although why this would appear only in the Chinese group is not clear. Prior research does provide some possible explanations for the finding. In particular, Chinese individuals (and people of East Asian and South Asian descent broadly) have been shown to have a predisposition to the atherogenic and diabetogenic effects of the metabolic syndrome at lower cutoff points of waist circumference, fasting plasma glucose levels, and most notably, triglycerides compared to other racial or ethnic groups.^25^^,^^46^ Furthermore, it is known that the primary adverse lipogenic effect of HRT is to increase triglyceride levels significantly.^11^^,^^47^ Since Chinese individuals are known to have higher triglycerides than White individuals and potentially be predisposed to the negative cardiovascular effects of triglyceride levels at lower levels,^48^, ^49^, ^50^, ^51^, ^52^, ^53^ it may be that the increase in triglycerides from HRT may be particularly harmful in Chinese adults. It also may be the case that currently unrecognized confounders underly our findings. Importantly, self-reported race and ethnicity is a social categorization and does not necessarily entail genetic ancestry.^54^ However, within the MESA study, self-reported race and ethnicity has been shown to be a strongly correlated proxy to genetic ancestry, particularly among the White, Black, and Chinese groups, and so may serve as an indirect marker for relevant differences in genetics.^55^, ^56^, ^57^ Overall, additional analyses in East Asian cohorts are needed to reproduce our finding.

### Study Limitations

There are a number of both strengths and limitations to the current study. Strengths include that our study population had high HRT use, as enrollment occurred immediately before the seminal WHI study that dramatically reduced HRT use, and that participants were fairly well divided in starting HRT close to the time of menopause or more than 5 years after menopause. This made our study population well suited to study the timing hypothesis. In addition, we have longer-term cardiovascular outcome data than most prior studies of HRT, extending up to 16 years, and we have robust data on cardiovascular outcomes, all-cause mortality, and cardiac risk factors. Our study population was also intentionally designed to include ethnic and racial diversity. However, there were also limitations to this study. First, our data are observational rather than randomized, and so should be regarded as exploratory and hypothesis-generating. Second, we did not have comprehensive documentation of HRT formulations that participants were on or consistent follow-up data on how their HRT use changed. However, the lack of granular data on new HRT initiation and HRT follow-up would be expected to introduce noise and bias towards the null. Given limitations in data availability, we are unable to determine if a particular HRT formulation is preferable to another for CVD prevention, or if there is an optimal duration of HRT use. Some degree of recall bias is also likely among participants, particularly older participants, in remembering the age of menopause and HRT initiation. This would be expected to lead to underreporting of HRT use among older patients, which could have introduced noise into the data but would be expected to bias towards negative results for the timing hypothesis. Our cohort also excluded participants with existing CVD, and so could only investigate HRT effects as primary CVD prevention. The HRT group also notably had significantly better cardiovascular health markers with lower BMI, cholesterol levels, and CAC scores, and so results may have been subject to a healthy-user bias, although multivariate modeling was used to limit this effect. Finally, there is some evidence that the first year of HRT has the highest risk of adverse cardiovascular and thromboembolic outcomes, with subsequent years having lower or reduced risk, and the majority of our participants (83%) had been on HRT for 1 year or more.^58^^,^^59^ This may have provided a bias towards the finding of a protective effect of HRT.

## Conclusions

We found that HRT use initiated within 5 years of natural menopause was associated with lower MACE and death across racial and ethnic groups, with the exception of a hypothesis-generating finding of a higher risk of adverse outcomes among Chinese women with the metabolic syndrome or with elevated triglycerides. Taken together, these findings lend additional support to the “timing hypothesis” that early initiation of HRT close to the time of menopause may decrease CVD risk and death across diverse populations. Further translational efforts are needed to validate the unique finding of increased risk of HRT in Chinese women with metabolic risk factors. Importantly, HRT use is not currently recommended for the primary prevention of CVD, and our present findings should be interpreted as hypothesis-generating given the limitations of observational study designs. Our findings also provide some support for the theory that atherogenic risk factors, particularly elevated triglycerides and the presence of metabolic syndrome, may underly the relationship between HRT and worse CVD outcomes, specifically in women of Chinese ethnicity. Overall, our findings add to the complex literature around the use of HRT for the primary prevention of CVD, emphasizing the need for an individualized approach to the initiation of HRT in patients undergoing natural menopause.

## Funding support and author disclosures

The MESA study was supported by contracts 75N92020D00001, HHSN268201500003I, N01-HC-95159, 75N92020D00005, N01-HC-95160, 75N92020D00002, N01-HC-95161, 75N92020D00003, N01-HC-95162, 75N92020D00006, N01-HC-95163, 75N92020D00004, N01-HC-95164, 75N92020D00007, N01-HC-95165, N01-HC-95166, N01-HC-95167, N01-HC-95168, and N01-HC-95169 from the 10.13039/100000050National Heart, Lung, and Blood Institute, and by grants UL1-TR-000040, UL1-TR-001079, and UL1-TR-001420 from the 10.13039/100006108National Center for Advancing Translational Sciences (NCATS). Study sponsors were not involved in study design, data interpretation, writing, or the decision to submit the article for publication. The funders had no role in the design and conduct of the study; collection, management, analysis, and interpretation of the data; preparation, review, or approval of the article; and decision to submit the article for publication. The authors have reported that they have no relationships relevant to the contents of this paper to disclose.
