# Supplementary material for: Hormone Replacement Therapy and Cardiovascular Outcomes by Race and Ethnicity: MESA (Multi-Ethnic Study of Atherosclerosis)
Source: JACC Adv. 2026 Jan 23;5(2):102561. doi: 10.1016/j.jacadv.2025.102561 (PMC12860954; doi:10.1016/j.jacadv.2025.102561)
Supplement: Supplemental material [file mmc1.docx]

**SUPPLEMENTAL MATERIAL**

*Racial/ethnicity and cardiovascular and mortality outcomes with hormone therapy: the Multi-Ethnic Study of Atherosclerosis*

Spencer Flynn, MD, Massachusetts General Hospital; Amier Haidar, MD, UCLA Department of Medicine; Icy Liang, BS, Brown University; Karol Watson, MD, PhD, UCLA Division of Cardiology; Tamara Horwich, MD, MS, UCLA Division of Cardiology; Preethi Srikanthan, MD, MS, UCLA Division of Endocrinology

**SUPPLEMENTAL TABLES**

**Supplemental Table 1.** Hormone Replacement within 5 Years of Menopause and MACE/Death, Excluding Participants Within 5 Years of Menopause at Exam 1… page 3

**Supplemental Table 2.** Hormone Replacement and Adverse Cardiovascular Outcomes by MACE Category, with stratification by time of HRT initiation and by race … page 4

**Supplemental Table 3.** Hormone Replacement and Adverse Cardiovascular Outcomes by MACE Category, with Stratification by Age Participant Underwent Menopause … page 7

**Supplemental Table 4.** Demographics and Clinical Characteristics of Participants Stratified by use of Hormone Replacement Therapy in Imputed Full Cohort … page 9

**Supplemental Table 5.** Hormone Replacement and MACE/Death in Imputed Full Cohort … page 12

**Supplemental Table 6.** Hormone Replacement, Body Composition and Atherogenic Markers, and MACE/Death in Chinese Participants … page 14

**Supplemental Table 7.** Hormone Replacement and Interactions with the Metabolic Syndrome with MACE/Death, Stratified by Race and Ethnicity … page 17

**SUPPLEMENTAL FIGURES**

**Supplemental Figure 1:** Study Flowchart … page 20

| **Supplemental Table 1:** Hormone Replacement within 5 Years of Menopause and MACE/Death, Excluding Participants Within 5 Years of Menopause at Exam 1 | |
| --- | --- |
|  | HR (95% CI), P-value |
| MACE | 0.77 (0.60-0.97), p=0.030 |
| All-cause mortality | 0.73 (0.59-0.89), p=0.002 |
| MACE, within 5 years | 0.72 (0.54-0.97), p=0.031 |
| MACE, after 5 years | 0.90 (0.65-1.24), p=0.525 |
| All-cause mortality, within 5 years | 0.61 (0.47-0.80), p<0.001 |
| All-cause mortality, after 5 years | 0.90 (0.69-1.17), p=0.424 |
| All models included age, smoking status, diabetes, BMI, hypertension, physical activity, income, and LDL levels as covariates. | |

| **Supplemental Table 2:** Hormone Replacement and Adverse Cardiovascular Outcomes by MACE Category, with stratification by time of HRT initiation and by race | |
| --- | --- |
|  | HR (95% CI), P-value |
| No HRT | Ref |
| Myocardial infarction | 0.66 (0.42-1.05), p=0.080 |
| Heart failure | 0.61 (0.39-0.94), p=0.025 |
| Stroke | 0.74 (0.49-1.11), p=0.150 |
| Cardiac mortality | 0.72 (0.46-1.11), p=0.140 |
| Coronary revascularization | 0.67 (0.41-1.11), p=0.125 |
|  |  |
| **By time of initiation of HRT**  No HRT | Ref. |
| Myocardial infarction, within 5 years | 0.75 (0.44-1.28), p=0.288 |
| Myocardial infarction, after 5 years | 0.65 (0.33-1.28), p=0.211 |
| Heart failure, within 5 years | 0.63 (0.37-1.06), p=0.082 |
| Heart failure, after 5 years | 0.60 (0.32-1.14), p=0.110 |
| Stroke, within 5 years | 0.61 (0.36-1.03), p=0.065 |
| Stroke, after 5 years | 1.09 (0.66-1.82), p=0.731 |
| Cardiac mortality, within 5 years | 0.37 (0.18-0.73), p=0.005 |
| Cardiac mortality, after 5 years | 1.39 (0.83-2.32), p=0.210 |
| Revascularization procedure, within 5 years | 0.72 (0.41-1.28), p=0.265 |
| Revascularization procedure, after 5 years | 0.50 (0.21-1.18), p=0.112 |
|  |  |
| **By race** |  |
| No HRT | Ref. |
| Chinese, myocardial infarction | 5.64 (1.46-21.8), p=0.012 |
| Chinese, heart failure | 4.01 (1.19-13.5), p=0.025 |
| Chinese, stroke | Model violated assumptions |
| Chinese, cardiac mortality | 3.50 (0.82-14.9), p=0.090 |
| Chinese, coronary revascularization | 2.46 (0.51-11.8), p=0.263 |
|  |  |
| White, myocardial infarction | 0.60 (0.31-1.15), p=0.122 |
| White, heart failure | 0.56 (0.30-1.04), p=0.066 |
| White, stroke | 1.16 (0.61-2.17), p=0.651 |
| White, cardiac mortality | 0.70 (0.33-1.46), p=0.342 |
| White, coronary revascularization | 0.84 (0.45-1.59), p=0.592 |
|  |  |
| Black, myocardial infarction | 0.30 (0.09-1.07), p=0.064 |
| Black, heart failure | 0.30 (0.10-0.87), p=0.026 |
| Black, stroke | 0.43 (0.17-1.07), p=0.069 |
| Black, cardiac mortality | 0.46 (0.20-1.05), p=0.066 |
| Black, coronary revascularization | Model violated assumptions |
|  |  |
| Hispanic, myocardial infarction | 0.36 (0.10-1.29), p=0.116 |
| Hispanic, heart failure | 0.68 (0.20-2.26), p=0.529 |
| Hispanic, stroke | 0.57 (0.24-1.35), p=0.205 |
| Hispanic, cardiac mortality | 0.59 (0.19-1.8), p=0.350 |
| Hispanic, coronary revascularization | 0.25 (0.05-1.27), p=0.095 |
| All models included age, smoking status, diabetes, BMI, hypertension, physical activity, income, and LDL levels as covariates, with HRT use as the independent variable and the labeled outcome (e.g. myocardial infarction) as the dependent variable.  Reference group was always no HRT. In the race/ethnic groups, reference group was no HRT within that race/ethnic group. There were 1,268 individuals without prior HRT use, 1,159 total individuals with prior HRT use, and 751 with HRT use within 5 years. By ethnicity, there were 934 total white participants (596 with prior HRT), 304 Chinese participants (102 with prior HRT), 648 Black participants (259 with prior HRT), and 541 Hispanic participants (202 with prior HRT).  Resuscitated cardiac arrest had insufficient events for independent analysis (n=12 in full cohort) and so was omitted. | |

| **Supplemental Table 3:** Hormone Replacement and MACE/death, with Stratification by Age Participant Underwent Menopause | |
| --- | --- |
|  | HR (95% CI), P-value |
| No HRT | Ref |
| MACE, below median age menopause | 0.82 (0.60-1.13), p=0.230 |
| MACE, above median age menopause | 0.67 (0.47-0.94), p=0.020 |
| Mortality, below median age menopause | 0.72 (0.55-0.96), p=0.023 |
| Mortality, above median age menopause | 0.92 (0.53-0.97), p=0.029 |
|  |  |
| **By time of HRT initiation**  No HRT | Ref. |
| MACE, below median age menopause and HRT within 5 years | 0.75 (0.51-1.12), p=0.157 |
| MACE, below median age menopause and HRT after 5 years | 1.04 (0.69-1.58), p=0.854 |
| MACE, above median age menopause and HRT within 5 years | 0.68 (0.45-1.01), p=0.056 |
| MACE, above median age menopause and HRT after 5 years | 0.70 (0.42-1.15), p=151 |
| All-cause mortality, below median age menopause and HRT within 5 years | 0.54 (0.37-0.78), p=0.001 |
| All-cause mortality, below median age menopause and HRT after 5 years | 1.02 (0.72-1.45), p=0.926 |
| All-cause mortality, above median age menopause and HRT within 5 years | 0.72 (0.50-1.03), p=0.070 |
| All-cause mortality, above median age menopause and HRT after 5 years | 0.76 (0.50-1.15), p=0.192 |
| All models included age, smoking status, diabetes, BMI, hypertension, physical activity, income, and LDL levels as covariates.  Reference group was always never HRT users. Pinteraction terms for all stratified comparisons were P>0.05, whether modeling age of menopause as a continuous variable or as a categorical variable of median age of menopause. Median age of menopause was 50. There were 1226 individuals who were age 50 or older at menopause (573 had prior HRT use, with 412 starting HRT within 5 years of menopause), and 1201 individuals less than 50 at menopause (586 had prior HRT use, with 339 starting HRT within 5 years of menopause). | |

| **Supplemental Table 4:** Demographics and Clinical Characteristics of Participants Stratified by Use of Hormone Replacement Therapy in Imputed Full Cohort | | | |
| --- | --- | --- | --- |
|  | **No hormone replacement, n=1351** | **Received hormone replacement, n=1222** | **P-value** |
| Age, years | 65.7 (9.5) | 63.5 (8.7) | <0.001 |
| Age started hormone replacement | NA | 52.2 (8.6) | NA |
| Using HRT at Exam 1 | NA | 730 (59.7%) | NA |
| Using HRT at Exam 4 | NA | 194 (15.9%) | NA |
| Years of HRT by Exam 1, median (IQR) | NA | 5 (1-12) | NA |
| Race or ethnicity  White  Chinese  Black  Hispanic | 360 (26.46%)  210 (15.5%)  425 (31.5%)  356 (26.4%) | 619 (50.7%)  104 (8.5%)  285 (23.3%)  214 (17.5%) | <0.001 |
| Weight, kg | 73.0 (17.0) | 72.4 (15.6) | 0.370 |
| Body mass index | 29.1 (6.3) | 28.0 (5.6) | <0.001 |
| Waist circumference, cm | 98.6 (15.6) | 95.6 (15.1) | <0.001 |
| Waist hip ratio | 0.919 (0.081) | 0.894 (0.083) | <0.001 |
| Systolic blood pressure, mmHg | 131.5 (23.9) | 126.7 (22.6) | <0.001 |
| Low-density lipoprotein, mg/dL, median (IQR) | 121 (100 -143) | 113 (94-134) | <0.001 |
| High-density lipoprotein, mg/dL, median (IQR) | 51 (44 - 62) | 57(47-68) | <0.001 |
| Triglycerides, mg/dL, median (IQR) | 110 (80-157.5) | 115(79-166.0) | 0.2 |
| C-reactive protein, mg/dL, median (IQR) | 2.3 (1.0-4.9) | 2.9 (1.1-5.9) | 0.002 |
| Creatinine, mg/dL, median (IQR) | 0.8 (0.7-0.9) | 0.8 (0.7-0.9) | 0.8 |
| Coronary artery calcium, Agatston, median (IQR) | 0.0 (0.0-63.6) | 0.0 (0.0-4.6) | 0.002 |
| Hypertension | 705 (52.2) | 593 (48.5) | 0.070 |
| Diabetes, yes | 162 (12.0%) | 115 (9.4%) | 0.107 |
| Metabolic syndrome, yes | 604 (44.9%) | 4447 (36.7%) | <0.001 |
| Prior pregnancy, yes | 1200 (88.8%) | 1074 (87.9%) | 0.498 |
| Cancer, yes | 118 (8.7%) | 111 (9.1%) | 0.547 |
| Smoking  Previous  Current | 337 (25.0%)  145 (10.8%) | 422 (34.7%)  138 (11.3%) | <0.001 |
| Exercise, MET minutes per week | 348 (153- 660) | 366 (192-645) | 0.033 |
| Prior oophorectomy | 145 (10.7%) | 242 (19.8%) | <0.001 |
| Prior hysterectomy | 294 (21.8%) | 454 (37.2%) | <0.001 |
| Hormone replacement type  Estrogen only  Estrogen and progesterone  Not recorded | NA | 432 (35.4%)  356 (29.1%)  434 (35.5%) | NA |
| Income scale | 6.8 (3.5) | 8.5 (3.5) | <0.001 |
| Started hormone replacement within 5 years of menopause | NA | 826 (67.6%) | NA |
| MACE or cardiac death | 209 (15.5%) | 138 (11.3%) | 0.002 |
| All-cause mortality, | 295 (21.8%) | 179 (14.6%) | <0.001 |
| Cardiac death | 71 (5.3%) | 38 (3.1%) | 0.009 |
| MACE, excluding cardiac death | 178 (13.2%) | 120 (9.8%) | 0.009 |
| Myocardial infarction | 55 (4.1%) | 30 (2.5%) | 0.029 |
| Stroke | 72 (5.3%) | 44 (3.6%) | 0.044 |
| *Abbreviations:* HRT=hormone replacement therapy, MET = metabolic equivalent of task  All numeric variables are presented as mean (SD) unless otherwise specified. All categorical variables are presented as count (percentage). | | | |

| **Supplemental Table 5:** Hormone Replacement and MACE/Death in Imputed Full Cohort | | |
| --- | --- | --- |
|  | HR (95% CI) | P-value |
| No HRT | Ref | NA |
| All-cause mortality | 0.73 (0.60-0.89) | 0.002 |
| MACE | 0.78 (0.62-0.98) | 0.035 |
| All-cause mortality, HRT within 5 years of menopause | 0.61 (0.47-0.78) | 0.001 |
| MACE, HRT within 5 years of menopause | 0.71 (0.54-0.94) | 0.018 |
| All-cause mortality, after 5 years of menopause | 0.94 (0.72-1.21) | 0.606 |
| MACE, after 5 years of menopause | 0.97 (0.72-1.32) | 0.847 |
| **Race Models** |  |  |
| No HRT within same race/ethnic group | Ref | NA |
| White, MACE | 0.79 (0.56-1.12) | 0.187 |
| White, mortality | 0.65 (0.48-0.89) | 0.008 |
| Black, MACE | 0.55 (0.34-0.91) | 0.023 |
| Black, mortality | 0.66 (0.46-0.96) | 0.031 |
| Hispanic, MACE | 0.47 (0.26-0.86) | 0.017 |
| Hispanic, mortality | 0.63 (0.47-1.06) | 0.088 |
| Chinese, MACE | 2.34 (1.11-4.91) | 0.036 |
| Chinese, mortality | 1.46 (0.81-2.62) | 0.217 |
| **Chinese models with metabolic syndrome or triglycerides** |  |  |
| No HRT in Chinese group | Ref | NA |
| MACE, HRT and no metabolic syndrome | 0.73 (0.20-2.70) | 0.673 |
| Mortality, HRT and no metabolic syndrome | 0.70 (0.24-2.05) | 0.534 |
| MACE, HRT and metabolic syndrome | 4.08 (1.47-11.3) | 0.026 |
| Mortality, HRT and metabolic syndrome | 2.48 (1.13-5.45) | 0.035 |
| MACE, HRT and low triglycerides | 1.28 (0.47-3.44) | 0.642 |
| Mortality, HRT and low triglycerides | 0.62 (0.26-1.49) | 0.300 |
| MACE, HRT and high triglycerides | 5.92 (1.59-22.01) | 0.005 |
| Mortality, HRT and high triglycerides | 3.54 (1.47-8.51) | 0.013 |
| All models controlled for **age, income level, smoking status, diabetes, hypertension, physical activity, and LDL levels.** Metabolic syndrome was ascertained according to the National Cholesterol Education Program 2004 guidelines and was applied equivalently across racial groups. Elevated triglycerides was defined as triglycerides$\geq$150. The triglycerides variable used in the individual model was continuous as mg/dL of triglycerides. Imputed dataset had n=2573 individuals with no missing covariates.  *Abbreviations:* HRT = hormone replacement therapy, BMI = body mass index, WHR = waist-hip ratio, WC = waist circumference, FGL = fasting glucose level, CRP = C-reactive protein | | |

| **Supplemental Table 6:** Hormone Replacement, Body Composition and Atherogenic Markers, and MACE/Death in Chinese Participants | | |
| --- | --- | --- |
|  | HR (95% CI) | P-value |
| **MACE Models** |  |  |
| BMI model and MACE:  HRT  BMI  HRT:BMI interaction | 2.27 (1.06-4.87)  1.06 (0.94-1.17)  1.01 (0.81-1.26) | 0.035  0.121  0.940 |
| WHR model and MACE:  HRT  WHR  HRT:WHR interaction | 2.36 (1.09-5.11)  1.61 (0.88-2.93)  2.02 (0.56-7.32) | 0.029  0.119  0.282 |
| Waist circumference model and MACE:  HRT  WC  HRT:WC interaction | 2.47 (1.14-5.37)  1.04 (1.01-1.08)  1.03 (0.95-1.11) | 0.022  0.024  0.466 |
| Fasting glucose and MACE:  HRT  FGL  HRT:FGL interaction | 4.22 (0.27-64.8)  1.01 (1.00-1.020)  0.99 (0.97-1.02) | 0.302  0.187  0.630 |
| Triglycerides and MACE:  HRT  Triglycerides  HRT:triglycerides interaction | 0.28 (0.05-1.63)  0.99 (0.98-1.00)  1.01 (1.00-1.03) | 0.263  0.052  0.015 |
| Metabolic syndrome and MACE:  HRT  Metabolic syndrome  HRT: metabolic syndrome interaction | 0.90 (1.11-3.00)  0.32 (0.09-1.09)  5.40 (1.09-26.95) | 0.869  0.070  0.040 |
| CRP and MACE:  HRT  CRP  HRT: CRP interaction | 2.20 (1.03-4.724)  1.00 (0.88-1.12)  0.84 (0.57-1.23) | 0.032  0.944  0.368 |
| CAC and MACE:  HRT  CAC  HRT:CAC interaction | 1.66 (0.74-3.76)  1.00 (1.00-1.00)  1.00 (1.00-1.00) | 0.045  0.015  0.658 |
| **All-cause Mortality Models** |  |  |
| BMI model and all-cause mortality:  HRT  BMI  HRT:BMI interaction | 1.34 (0.73-2.47)  1.03 (0.95-1.11)  0.98 (0.81-1.19) | 0.348  0.517  0.843 |
| WHR model and all-cause mortality:  HRT  WHR  HRT:WHR interaction | 1.35 (0.73-2.49)  1.04 (0.66-1.62)  0.70 (0.28-1.77) | 0.339  0.879  0.449 |
| Waist circumference model and all-cause mortality:  HRT  WC  HRT:WC interaction | 1.34 (0.73-2.48)  1.01 (0.98-1.04)  1.00 (0.94-1.06) | 0.348  0.390  0.939 |
| Fasting glucose and all-cause mortality:  HRT  FGL  HRT:FGL interaction | 0.33 (0.03-3.63)  1.00 (0.98-1.02)  1.01 (0.99-1.04) | 0.365  0.792  0.232 |
| Triglycerides and all-cause mortality:  HRT  Triglycerides  HRT:triglycerides interaction | 0.26 (0.06-1.06)  1.00 (0.99-1.00)  1.01 (1.00-1.02) | 0.060  0.102  0.009 |
| Metabolic syndrome and all-cause mortality:  HRT  Metabolic syndrome  HRT: metabolic syndrome interaction | 0.76 (0.27-2.15)  0.97 (0.41-2.30)  2.52 (0.68-9.34) | 0.609  0.945  0.167 |
| CRP and all-cause mortality:  HRT  CRP  HRT: CRP interaction | 1.34 (0.73-2.46)  0.96 (0.85-1.09)  0.75 (0.43-1.32) | 0.353  0.565  0.315 |
| CAC and all-cause mortality:  HRT  CAC  HRT:CAC interaction | 1.24 (0.66-2.36)  1.00 (1.00-1.00)  1.00 (1.00-1.00) | 0.504  0.327  0.371 |
| All models controlled for **age, income level, smoking status, diabetes, hypertension, physical activity, and LDL levels.** Metabolic syndrome was ascertained according to the National Cholesterol Education Program 2004 guidelines and was applied equivalently across racial groups. Elevated triglycerides was defined as triglycerides$\geq$150. The triglycerides variable used in the individual model was continuous as mg/dL of triglycerides.  *Abbreviations:* HRT = hormone replacement therapy, BMI = body mass index, WHR = waist-hip ratio, WC = waist circumference, FGL = fasting glucose level, CRP = C-reactive protein | | |

| **Supplemental Table 7:** Hormone Replacement and Interactions with the Metabolic Syndrome with MACE/Death, Stratified by Race and Ethnicity | | |
| --- | --- | --- |
|  | MACE  HR (95% CI) | Mortality  HR (95% CI) |
| White  HRT  Metabolic syndrome  HRT:metabolic syndrome | 0.78 (0.48-1.25)  1.35 (0.78-2.32)  1.01 (0.52-1.97) | 0.62 (0.40-0.95)  1.42 (0.89-2.27)  1.10 (0.61-1.99) |
| White  HRT  Triglycerides  HRT:triglycerides | 0.88 (0.57-1.26)  1.42 (0.84-2.40)  0.69 (0.35-1.38) | 0.65 (0.44-0.95)  1.16 (0.74-1.81)  0.92 (0.50-1.72) |
| White  HRT  BMI  HRT:BMI | 0.77 (0.55-1.11)  1.01 (0.98-1.04)  1.00 (0.94-1.06) | 0.65 (0.48-0.89)  1.02 (0.99-1.05)  0.99 (0.94-1.05) |
| Black  HRT  Metabolic syndrome  HRT:metabolic syndrome | 0.45 (0.19-1.05)  1.66 (0.92-2.98)  1.26 (0.43-3.72) | 0.75 (0.45-1.26)  1.10 (0.71-1.71)  0.72 (0.33-1.57) |
| Black  HRT  Triglycerides  HRT:triglycerides | 0.55 (0.31-0.98)  0.99 (0.47-2.05)  0.71 (0.17-2.96) | 0.69 (0.45-1.04)  1.09 (0.61-1.94)  0.69 (0.24-1.97) |
| Black  HRT  BMI  HRT:BMI | 0.52 (0.31-0.88)  1.01 (0.98-1.05)  0.97 (0.89-1.07) | 0.65 (0.44-0.95)  1.01 (0.98-1.04)  1.04 (0.98-1.11) |
| Hispanic  HRT  Metabolic syndrome  HRT:metabolic syndrome | 0.87 (0.40-1.90)  1.43 (0.81-2.54)  0.44 (0.16-1.24) | 0.74 (0.34-1.63)  1.19 (0.69-2.04)  0.82 (0.29-2.31) |
| Hispanic  HRT  Triglycerides  HRT:triglycerides | 0.52 (0.26-1.06)  0.60 (0.34-1.05)  0.89 (0.25-3.11) | 0.62 (0.32-1.19)  0.79 (0.46-1.34)  1.20 (0.42-3.42) |
| Hispanic  HRT  BMI  HRT:BMI | 0.50 (0.28-0.91)  1.06 (1.02-1.11)  0.99 (0.89-1.10) | 0.66 (0.39-1.12)  1.00 (0.96-1.05)  1.00 (0.90-1.11) |
| Chinese  HRT  Metabolic syndrome  HRT:metabolic syndrome | 0.90 (1.11-3.00)  0.32 (0.09-1.09)  5.40 (1.09-26.95) | 0.76 (0.27-2.15)  0.97 (0.41-2.30)  2.52 (0.68-9.34) |
| Chinese  HRT  Triglycerides  HRT:triglycerides | 0.28 (0.05-1.63)  0.99 (0.98-1.00)  1.01 (1.00-1.03) | 0.26 (0.06-1.06)  1.00 (0.99-1.00)  1.01 (1.00-1.02) |
| Chinese  HRT  BMI  HRT:BMI | 2.27 (1.06-4.87)  1.06 (0.97-1.17)  1.01 (0.81-1.26) | 1.34 (0.73-2.47)  1.03 (0.95-1.11)  0.98 (0.81-1.19) |
| All models included age, smoking status, diabetes, BMI, hypertension, physical activity, income, and LDL levels as covariates. Metabolic syndrome was ascertained according to the National Cholesterol Education Program 2004 guidelines and was applied equivalently across racial groups. Within the metabolic syndrome, triglycerides was defined as triglycerides$\geq$150. The triglycerides variable used in the individual model was continuous as mg/dL of triglycerides. | | |

6814 MESA Exam1 participants

4241 excluded:

- 3213 men
- 329 without data on HRT use
- 271 women stating premenopausal, or had period in last 12 months
- 414 women with surgical menopause
- 146 with missing covariates
  - 105 missing income
  - 39 missing LDL
  - 1 missing exercise
  - 1 missing diabetes
- 14 without outcome data

2427 participants included in final study population

1268 participants never used HRT

1159 participants prior or current HRT

##

**Supplemental Figure 1:** Study Flowchart
